# Complete plastid genome of the endangered species *Paraisometrum mileense* (Gesneriaceae) endemic to China

**DOI:** 10.1080/23802359.2019.1677186

**Published:** 2019-10-15

**Authors:** Jing Meng, Linna Zhang, Jun He

**Affiliations:** a College of Horticulture and Landscape, Yunnan Agricultural University, Kunming, China;; b Kunming Institute of Botany, Chinese Academy of Sciences, Kunming, China

**Keywords:** *Paraisometrum mileense*, plastid genome, endangered species, phylogenetic analysis

## Abstract

*Paraisometrum mileense* is a unique species of *Paraisometrum* endemic to south-west China, which is a “100-years-lost” plant and was rediscovered in 2006. In this paper, complete plastid genome of *P. mileense* is firstly assembled and characterized. The length of total plastid genome is 153,259 bp consisting of a large single-copy region (LSC) of 84,293 bp, a small single-copy region (SSC) of 18,162 bp, and two inverted repeat regions (IRs) of 25,402 bp. In total, 113 genes are predicted, including 80 protein-coding genes (PCGs), four rRNA genes and 29 tRNA genes. Phylogenetic analysis indicated that *P. mileense* with the other eight Gesneriaceae species formed a clade with a 100% bootstrap support.

*Paraisometrum* W. T. Wang is a monotypic genus, including *P*. *mileense* W. T. Wang, which is endemic to south-west China (Weitzman et al. 1997; Wang et al. 1998). This species had been considered as extinct for 100 years. From 2006, at least three wild populations were continuously rediscovered in Yunnan, Guangxi and Guizhou Province, respectively (Shui 2007; Xu et al. 2009; Gao and Xu 2011; Chen et al. 2014)). According to its extremely small number of wild individuals (totally 840 mature plants) and vulnerability to human activities, *P. mileense* can be considered as an endangered (EN) species under the IUCN global species programme. The results of genetic diversity study and phylogenetic analysis indicated that *P. mileense* had relatively low levels of genetic diversity and indecisive systematic position (Weitzman et al. 1997; Tan et al. 2011; Möller et al. 2011; Chen et al. 2014). In this study, we present the complete plastid genome of *P. mileense* (GenBank accession number: MK342624) for the first time by using Illumina next-generation sequencing techniques and combining *de novo* assembly. This research will enrich and help to resolve the evolutionary relationship between *P*. *mileense* and other genus or species within Gesneriaceae in the future.

Total DNA was extracted from fresh leaves of *P. mileense* with a modified CTAB method (Doyle and Doyle 1987). The material was obtained from Shilin County, Yunnan (103°33′45″E, 24°36′35″N), and voucher specimens were deposited in the herbarium of KUN (voucher: SL001). After purified, the extracted DNA was sequenced using the Illumina Miseq platform (Illumina, San Diego, CA, USA). High-quality reads were assembled using CLC Genomic Workbench v 10 (CLC Bio., Aarhus, Denmark) with the default parameters. All contigs were selected by performing a BLAST checked against the reference genome of *Boea hygrometrica* (NC016468), the selected contigs were ordered and oriented according to the reference genome, and the gaps produced between contigs were filled and examined by mapping raw reads to the self-sequence. Annotation of the protein-coding genes (PCG), transfer RNAs (tRNAs) and ribosomal RNAs (rRNAs) was employed by the program DOGMA (Wyman et al. 2004), and then manually adjusted using Geneious v 8.0.2 (https://www.geneious.com/) (Kearse et al. 2012). To identify the phylogenetic position of *P. mileense*, maximum likelihood (ML) tree was performed based on nine complete plastid genome sequences of Gesneriaceae and other two Labiatae species as outgroups in MEGA v 7.0 with 1000 rapid bootstrap replicates (Kumar et al. 2016).

The complete plastid genome of *P. mileense* is 153,259 bp, and exhibits a typical quadripartite structure found in most land plants which is made up of a large single-copy region (LSC) of 84,293 bp, a small single-copy region (SSC) of 18,162 bp, isolated by a pair of identical inverted repeat regions (IRs) of 25,402 bp. The total GC content of the whole sequence is 37.4%, gene annotation reveals that the complete plastid genome encodes 113 genes, including 80 protein-coding genes (PCGs), four ribosomal RNA (rRNA) genes and 29 transfer RNA (tRNA) genes. Among them, 18 intron-containing genes were investigated: two of which (*ycf3* and *clpP*) contained two introns, one of which (*rps12*) contained three exons, and the other 15 genes contained one intron. The *rps19* and *ycf1* locate at the junction of LSC/IRa and SSC/IRb, respectively. The phylogenetic analysis showed that *P. mileense* clustered with the other eight Gesneriaceae species, with a bootstrap support value of 100% (Figure 1).

**Figure 1.**
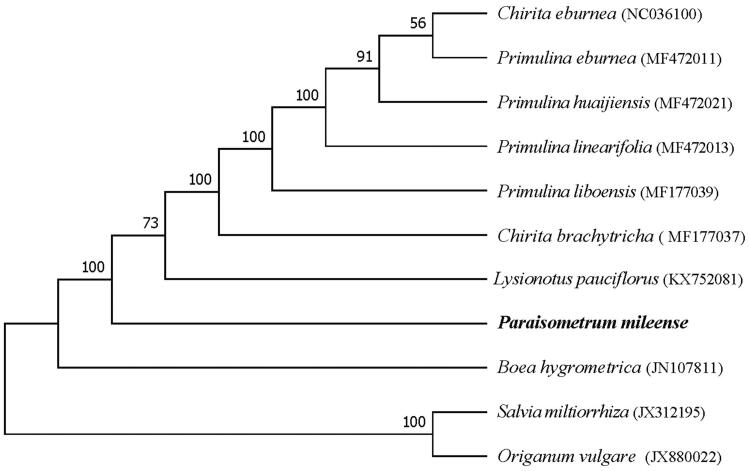
Maximum likelihood tree base on 11 species complete plastid genome sequences. Bootstrap support values are shown on each node based on 1000 replicates.
